# Initiation of maintenance hemodialysis through central venous catheters: study of patients' perceptions based on a structured questionnaire

**DOI:** 10.1186/s12882-019-1422-y

**Published:** 2019-07-17

**Authors:** Tanya T. Tang, Murray L. Levin, Shubhada N. Ahya, Khaled Boobes, Muhammad H. Hasan

**Affiliations:** 1 Division of Nephrology/Hypertension, Department of Medicine, Northwestern University Feinberg School of Medicine and Northwestern Memorial Hospital, Chicago, IL 60611 USA; 2 Present address: Foothills Nephrology, 126 Dillon Drive, Spartanburg, SC 29307 USA; 3 Present address: Nephrology Division OSU, 95 W 12th Ave#7, Columbus, OH 43210 USA; 4 United Elite Hospitalists, 12632 S Harlem Ave, Palos Heights, IL 60463 USA; 5 Highland Park, USA

**Keywords:** Hemodialysis, Tunneled catheters, Surgical referrals

## Abstract

**Background:**

Despite well-publicized suggestions to utilize arteriovenous fistulae and grafts to initiate hemodialysis, too many patients in the United States start dialysis via central venous catheters despite their well-known association with increased morbidity, mortality, and cost.

**Methods:**

To determine the reasons for this high rate of catheter use, and, ultimately, ways to reduce it, we developed a questionnaire designed to determine where in the process of patient care the process to fistula or graft placement was not completed, thus requiring the use of central venous catheters. The questionnaire was reviewed by several nephrologists not involved with the study. We administered the questionnaire to 52 consecutive hospitalized patients who started maintenance dialysis with catheters at a University-affiliated Hospital and referral center. The questionnaire asked each patient to provide details pertaining to pre-dialysis care, referrals, and follow-through on recommended referrals. If the patient did not see the physician to whom he/she was referred, we asked the reason(s) for such failure.

**Results:**

Patient responses showed that there were two major lapses in the transition from diagnosis of advanced kidney disease to construction of appropriate dialysis access: failure by the patients to see a nephrologist and/or an access surgeon, and failure by physicians to refer patients to an access surgeon. Twenty percent of the patients failed to follow up with either a nephrologist or a surgeon. Only 38% (15/40) of those seen by a nephrologist had been referred to a surgeon.

**Conclusions:**

The quality of care was impaired by lack of referral to surgeons by nephrologists and by lack of follow-through by patients. Areas for improvement include improved communications between physicians and patients and more careful follow-up by both physicians and patients. Several methods of providing better patient care and communication between patients and nephrologists are recommended.

**Electronic supplementary material:**

The online version of this article (10.1186/s12882-019-1422-y) contains supplementary material, which is available to authorized users.

## Background

The first-year mortality and morbidity rates encountered by maintenance hemodialysis patients in the United States are very high in comparison to the rest of the industrialized world [1]. In addition, continued use of a central venous catheter (CVC) for hemodialysis is associated with worse health, worse emotional well-being, diminished social functioning, less energy, poor sleep, a higher burden of kidney disease on daily life, and an overall lower quality of life compared to other access modalities [2]. There are well-documented increases in morbidity, mortality, and financial cost associated with catheter utilization compared to the use of fistulae and grafts [3–7], and infections are a leading cause of these catheter-induced complications [8, 9].

Despite these documented complications, as of 2013, 80% of patients in the United States initiated chronic hemodialysis using a catheter although some had maturing fistulae [10]. Even six months after the start of maintenance hemodialysis, more than 50% of patients were dialyzing through catheters [10].

Reasons for the high rate of use of catheters and underutilization of fistulae and grafts have been speculative. Possibilities include lack of sufficient numbers of pre-ESRD clinics, late referral to nephrologists [5, 6, 11–14], reimbursement patterns [15], heterogeneity in patient population, geographics, socioeconomic status [16–19], and lack of education about or unsuccessful education about dialysis modalities to allow well-informed patient decisions. Patients’ concerns about other dialysis access modalities include negative emotional and body images, pain with cannulation of arteriovenous accesses [20], disruption of identity, mindset, and lifestyle [21], and inadequate emotional and psychosocial patient support. Although late referral to nephrologists has been thought to be a major cause for the high initial use of catheters, Lok and Foley have pointed out that 42% of patients seen by nephrologists for over a year still initiated dialysis with a catheter [6]. More recently, patients seen by nephrologists for 6–12 months had a 0.61 chance of starting dialysis with a fistula or graft compared with those seen for more than 12 months [10]. Therefore, there must be additional reasons for initiation of hemodialysis with a catheter other than prior lack of seeing a nephrologist. Without doubt, quality of care is impacted by the high usage of catheters.

We sought to determine why this high rate of catheter use occurred, and how such care could be improved in the pre-dialysis care of patients. We sought information about the sequence of care our patients followed prior to initiation with a catheter by asking them to complete a survey in the presence of one of the authors so that we could determine those times in their clinical course when the path to appropriate dialysis access broke down. We selected this method because, to our knowledge, results of patients’ own histories concerning their course to dialysis access had not been published. Once areas of deviation from the course to proper access placement could be identified, those areas could be targets for interventions to enhance the creation and use of fistulae and grafts at optimum times prior to dialysis initiation. The quality of patient care would be enhanced and the cost of care reduced. Such interventions could then be offered to the general medical community to enhance patient care, quality, and safety.

## Methods

The aim of the study was to determine the reason(s) that large numbers of patients initiate hemodialysis using tunneled CVCs. We felt that the information could best be obtained from the patients themselves.

We asked 52 consecutive hospitalized patients who were initiating maintenance hemodialysis in a University-affiliated hospital through a tunneled CVC between January, 2014, and July, 2015, to complete a questionnaire (Additional file 1: Table S1). The questionnaire was derived by author MLL with input from authors SNA and TTT and review and suggestions by other members of our Nephrology Division who did not take part in the study.

All patients were older than 18 years old. Patients who had acute kidney injury that required permanent renal replacement therapy during the same hospitalization were excluded. We also excluded any patient who was confused or unable to cooperate.

## Results

Twenty-eight male and 24 female patients were enrolled in the study. Their ages ranged from 39 to 90 years old. The mean age was 60 years ±15 (Standard Deviation). Twenty-nine of the 52 patients (56%) were African-American; 14/52 (27%) were Caucasians; 7/52 (13%) were Hispanic; and 2/52 (4%) were of Asian heritage. Causes of end-stage renal disease (ESRD) were: diabetes mellitus in 30; hypertension in 6; membranoproliferative glomerulonephritis in 2; focal sclerosing glomerulonephritis in 6; and 1 each of congestive heart failure, scleroderma, multiple myeloma, lupus erythematosus, IgA nephropathy, amyloidosis, transplant rejection, and unknown. Most of the patients with diabetes mellitus were hypertensive. The other major comorbidities are listed above. Although not all patients had previously been part of our system, the demographics were similar to those of our chronic dialysis population.

**Fig. 1:**
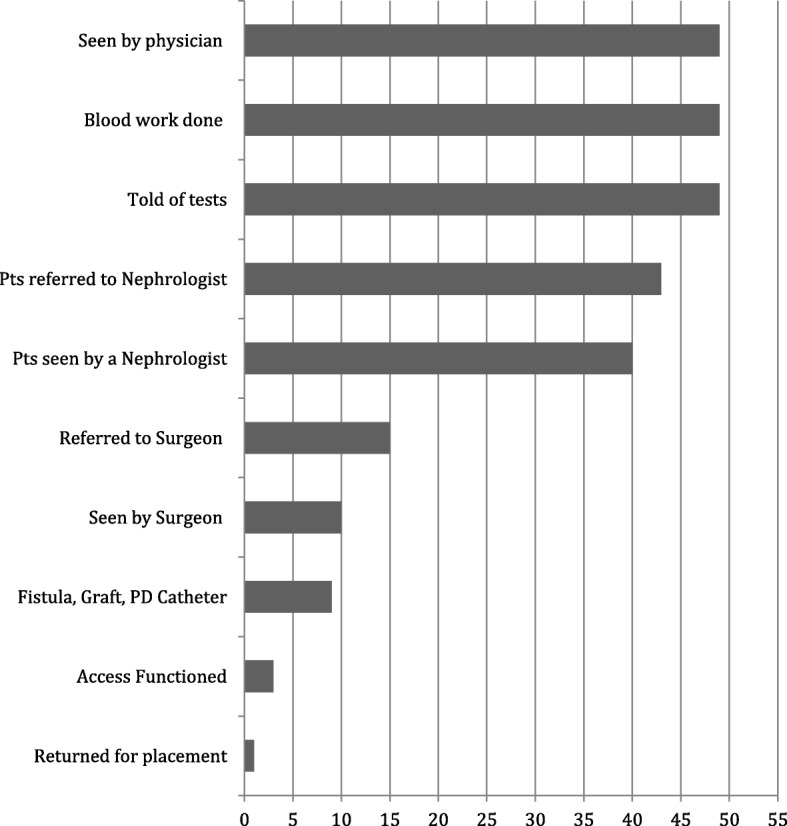
Results of Questionnaire Survey of Patients Starting Hemodialysis with a Central Venous Catheter. LEGEND: Each bar represents the number of patients in each category. Access Functioned refers to initial function that failed before dialysis was initiated

Ninety-four percent (49/52) of the total population had been seen by a physician in the preceding year, and all of those had been told that their kidney function was poor. Ninety-two percent (45/49) of those seen by a physician were referred to a nephrologist. Five of these did not follow up by seeing a nephrologist. Only 15 patients of the 40 who saw a nephrologist were referred to a surgeon for vascular access. Two patients were not referred for access because they wished to avoid the surgery while being evaluated for transplantation. The remainder of the patients who had not been referred could not give reasons for the late or absent referral although their nephrologists had discussed various types of access with several of them. Five patients did not comply with recommendations to see surgeons for access placement. Thus, ten patients (5 African-Americans, 3 Caucasians, 1 Hispanic, and 1 Asian) did not proceed with follow up to either a nephrologist or a surgeon. Reasons were that they feared surgery, forgot to schedule appointments, or did not feel ready for dialysis. One patient feared that he did not have sufficient insurance coverage. Of the 49 patients initiated on dialysis through a tunneled catheter and previously seen by a physician, only 15 had been referred to a surgeon for appropriate vascular access, only 10 actually saw the surgeon, and 7 had access placed. None of the patients was morbidly obese, a condition that may have precluded or made access surgery difficult or dangerous. Three of the placed accesses functioned initially but failed by the time dialysis was necessary. Our Medical Center is a referral institution, so the patients came from multiple sources in the Chicago Community. The results of the questionnaire are summarized in Fig. 1.

## Discussion

This study sought to determine why so many patients initiate hemodialysis using an inferior and more dangerous type of access, i.e. tunneled CVCs. Once the reason(s) for catheter use is/are determined, their use could be obviated by installing proper access well ahead of the clinically estimated time for initiation of maintenance hemodialysis. Our study demonstrates that, according to the patients, the largest drop-off in the progression of care to use of optimum dialysis access prior to the initiation of maintenance hemodialysis occurs between seeing a nephrologist and seeing a surgeon. Of the 49 patients who had previously seen any physician during the year prior to the initiation of dialysis through a tunneled catheter, only 10 had seen a surgeon for access, and only 7 had had an access placed. Obviously, none of those was ready for cannulation at the time dialysis was initiated.

There were several limitations to this study. It was performed in a single academic center in a large urban community. The results may not apply to patients in community hospitals, or in rural communities, or in many other countries. In addition, the study design could not discern whether some patients might have been reluctant to state that they had failed to follow through with recommendations, although some did. We were unable to verify the accuracy of patient responses, so patient statements or recall may not have been completely correct. We also did not determine the time duration between first seeing a physician and seeing a nephrologist. There may have been insufficient time for surgical referral. Patients with chronic kidney disease have higher mortality and increased hospitalizations when referred late to nephrologists [22]. Perhaps those unfortunate outcomes occurred because of the necessity to use catheters if there was insufficient time for construction of an arteriovenous fistula or placement of a graft prior to initiation of maintenance dialysis. Also, similar limitations on timing may have occurred if there was a sudden, unexpected loss of kidney function [6]. Regardless of the reasons, the fact that our patients experienced their initial hemodialysis using a catheter as access is irrefutable. Patient care quality was potentially impeded by lack of placement of optimal permanent access prior to initiation of maintenance dialysis.

Despite the Fistula First initiative [5, 6, 23, 24], hemodialysis catheter use for maintenance hemodialysis remains high and remains an ongoing issue in the United States [10]. We demonstrated that patient failure to comply and nephrologists’ failure to refer patients to surgeons for appropriate access placement were the two major reasons for the lack of availability of fistulae/grafts for access at the start of maintenance hemodialysis.

The reasons that patients gave for starting with a catheter emphasize the need for closer nephrologists’ follow-up and attention to the timing of access placement. Pisoni et al. [25] make a similar point about the need for reforms in pre-dialysis care. Either nephrologists were not referring patients to surgeons in an appropriate time frame, or they were not following up with patients to ensure that appropriate surgical consultation was obtained if there was sufficient time for referral. That the problems are not confined to our institution in particular and to the Chicago metropolitan area in general is given proof by the national data [10] and the data from Canada [26], both of which show large percentages of new dialysis patients starting with catheters.

Lopez-Vargas et al. [27] also concluded that late referral by nephrologists to surgeons was an underappreciated cause of initiation of maintenance dialysis with CVCs. However, they also pointed out that they did not interview patients and, therefore, could not determine patient factors in causing the use of catheters rather than fistulae or grafts. Our results are based on patient involvement through completion of our questionnaire. Additionally, as pointed out earlier in this manuscript, Lok and Foley [6] documented that catheters were still the initial access even when patients saw a nephrologist six-twelve months before dialysis initiation.

Having delineated the problem areas that lead to suboptimal care, we can offer many possible solutions toward the course of improvement. Although we did not investigate the efficacy of any of these possible actions, they appear to be logical suggestions to improve patient-doctor communications, follow-up, and follow-through. They provide a framework for policies to implement before dialysis is necessary to improve the dialytic care of patients with ESRD.

They include:

Using electronic medical record (EMR) to flag those patients who reach a predesignated estimated glomerular filtration rate (EGFR) to indicate the approaching need for access placement and ensure follow-up of patients by all appropriate members of the team of nephrologists, nurses, coordinators, surgeons and mental health specialists;

Veins in the non-dominant arm should be protected and venous mapping performed;

Patients should be referred to a vascular surgeon or interventional nephrologist at least 3 to 6 months before the anticipated start of dialysis;

Using the EMR to alarm when a referral has not been completed within a designated time;

Scheduling more frequent visits when higher Chronic Kidney Disease (CKD) Stages are reached so education about available therapeutic options may be given repeatedly to patients and their family members, enabling an appointment with a surgeon at the appropriate clinical stage;

Integrating CKD and ESRD care, including vascular access care under the umbrella of a comprehensive kidney care center;

Providing psychosocial and emotional support to allay fears and remove barriers to access placement;

Providing a peer support group to educate, encourage, and reinforce steps for access placement;

Having an access coordinator help patients understand the need for access while explaining the process in its various stages;

Having the coordinator assist patients with scheduling with surgeons, radiology, etc. by streamlining the process and minimizing the number of steps patients must take to obtain access;

Reminding patients to see the surgeon as often as such reminders are necessary;

Giving consideration to using an urgent start peritoneal program or early access grafts [28] if the fistula placement and maturation process would be too lengthy prior to the need for dialysis;

Using a graft rather than a fistula, especially in the elderly [29, 30];

Referring patients with failed transplants for access at appropriate times, the need for which was exemplified by one of our patients who required a catheter after her transplant had failed. Chan et al. have pointed out that nearly 2/3 of patients with failed renal transplants return to dialysis using catheters [31];

Medical Centers should provide ready access to motivated and well-trained surgeons for the placement of optimal arteriovenous fistulae, or early cannulation grafts, or laparoscopic placement of peritoneal dialysis catheters;

Continued emphasis should be placed on preservation of the integrity of peripheral and central veins in patients with CKD and limiting injury to these veins using the published American Society of Diagnostic and Interventional Nephrology guidelines [32].

In accordance with our suggestions for close follow-up and guidance, Fischer et al. pointed out that greater intensity of pre-dialysis nephrology care was associated with more favorable outcomes in older adults [33].

Finally, there has been recent doubt cast upon the superiority of fistulae and grafts vis-à-vis catheters in the elderly [34–40], and the question has been raised that patient characteristics such as severity of coexisting illness may have created selection bias in the use of catheters. Whether or not these questions regarding no difference in mortality attributable to catheter use in the elderly are definitive in providing support for the use of catheters, the frequency of infections, need for catheter exchange, and need for hospitalizations still exist. Increased cost and facility utilization still exist in patients dialyzed with central catheters, and these complications have been stressed again recently [41, 42]. Individualization of access, however, must be considered, but planning to avoid the need for catheters whenever possible must occur early, and close follow-up with patients must be stressed.

Our suggestions are summarized in Additional file 2.

## Conclusions

Regardless of the reasons, the fact that our patients experienced their initial hemodialysis using a catheter is irrefutable. Patient care quality was potentially impeded by lack of placement of optimal permanent access prior to initiation of maintenance dialysis. The major reasons for initiating dialysis with a CVC were patient and nephrologist delay in seeing surgeons for access placement. Whatever the underlying reasons for delay, nephrologists and primary care physicians must guide and compulsively encourage their patients with worsening chronic renal disease to see surgeons or interventional nephrologists for access and to keep appointments. Recurrent phone calls, emails, texting, or use of social media may be necessary. An interdisciplinary team (nurses, patient care coordinators) should be involved as well, along with preprogrammed reminders on hospital-patient computer communications systems to guide patients through the multistep pathway to obtaining appropriate permanent hemodialysis access. Such a course should mitigate the complications, excess mortality, and excess cost of care associated with the use of central venous catheters. The quality of patient care would be enhanced significantly.

## Additional files

Additional file 1: **Table S1.** Questionnaire on Hemodialysis Catheter use. (DOCX 95 kb)

Additional file 2: Suggestions for Expediting Appropriate Access Placement. (DOCX 12 kb)

## Data Availability

The datasets used and/or analyzed during this study are available from the corresponding author on reasonable request.

## Abbreviations

CKD: Chronic Kidney Disease
CVC: Central Venous Catheter
EGFR: Estimated Glomerular Filtration Rate
EMR: Electronic Medical Record
ESRD: End-Stage Renal Disease
PD: Peritoneal Dialysis
