# Supplementary material for: Initiation of maintenance hemodialysis through central venous catheters: study of patients' perceptions based on a structured questionnaire
Source: BMC Nephrol. 2019 Jul 17;20:270. doi: 10.1186/s12882-019-1422-y (PMC6637564; doi:10.1186/s12882-019-1422-y)
Supplement: Supplementary file 1 — Table S1. Questionnaire on Hemodialysis Catheter use. (DOCX 95 kb) [file 12882_2019_1422_MOESM1_ESM.docx]

**Additional file 1: Table S1**

**QUESTIONNAIRE ON HEMODIALYSIS CATHETER USE**

**Patient’s Name:**

**Age: Sex: Race:**

**Cause of ESRD:**

**Has the Patient Seen a Physician Within the Past Year? Y N**

**If So, Were Blood Tests Obtained? Y N**

**Was the Patient Ever Told That His/Her Kidney Function was Poor? Y N**

**Was the Patient Ever Referred to a Nephrologist (Kidney Doctor)? Y N**

**If So, Did the Patient See the Nephrologist? Y N**

**If Not, What Was the Reason for Not Seeing the Nephrologist: _______________________________________________________**

**Was the Patient Ever Referred to a Surgeon for Creation of a Fistula, Placement of a Graft, or Placement of a Peritoneal Dialysis (PD) Catheter? Y N**

**If So, Did the Patient See the Surgeon? Y N**

**If Not, What Was the Reason for Not Seeing the Surgeon:**

**________________________________________________________**

**Was a Fistula or Graft or PD Catheter Placement ever Attempted? Y N**

**If the Patient Saw the Surgeon, and No Fistula or Graft or PD Catheter Was Placed, What Was the Reason For Non-Placement:**

**____________________________________________________________**

**If a Fistula or Graft or PD Catheter Was Placed, Did It Function? Y N**

**If It Did Not Function, Was Another Attempt To Place One Made? Y N**
