# Supplementary material for: Initiation of maintenance hemodialysis through central venous catheters: study of patients' perceptions based on a structured questionnaire
Source: BMC Nephrol. 2019 Jul 17;20:270. doi: 10.1186/s12882-019-1422-y (PMC6637564; doi:10.1186/s12882-019-1422-y)
Supplement: Supplementary file 2 — Suggestions for Expediting Appropriate Access Placement. (DOCX 12 kb) [file 12882_2019_1422_MOESM2_ESM.docx]

**Additional file 2**

Electronic Medical Record Use:

Alarm to care team when EGFR reaches predetermined level requiring access placement

Alarm to care team when appointments regarding access placement are not kept

Care of arm to be used for dialysis:

Map veins and avoid use of veins in non-dominant arm.

Nephrology visits:

Schedule more frequent as EGFR falls

Have Chronic Kidney Disease Clinic next to Dialysis Clinic for patient teaching and acclimation

See transplant surgeon when appropriate if patient is a transplant candidate

Refer patient for access at least 3-6 months prior to anticipated start of dialysis

Access Coordinator:

Educate patient about process of access placement

Assist patient in navigating the process with radiology and surgery to expedite the process

Remind patient to comply with steps necessary for access placement as often as necessary to prevent patient from slipping through the cracks

Ensure follow-up with nephrology

New Advances or alternative actions to expedite non-catheter access:

Urgent start peritoneal dialysis

Early access grafts

Grafts, especially in the elderly

Failing Transplants:

Obtain appropriate access as early as necessary for smooth transition back to dialysis
